# More safer sex intervention needed for HIV-positive MSM with higher education level for prevention of sexually transmitted hepatitis C

**DOI:** 10.7448/IAS.17.4.19663

**Published:** 2014-11-02

**Authors:** Ada Wai Chi Lin, Ka Hing Wong, Kenny Chan

**Affiliations:** Special Preventive Programme, Department of Health, Kowloon, Hong Kong, China

## Abstract

**Introduction:**

The epidemiology of hepatitis C virus (HCV) infections in Chinese HIV-infected men who have sex with men (MSM) remains obscure. More data is required to understand the epidemic and set up preventive strategy.

**Materials and Methods:**

Baseline and annual testing of anti-HCV was in place for all HIV-infected MSM in the largest HIV clinic in Hong Kong. Logistic regression was used to compare those with HCV seroconversion (seroconverters) with those remained tested anti-HCV negative (non-seroconverters) to identify factors associated with incident HCV.

**Results:**

From 1999 to 2013, 1311 patients were tested for anti-HCV seroconversion, contributing to 6295 patient-years of observation. Fourteen (1.1%) patients seroconverted, with genotype 3 being most commonly detected. The overall incidence rate of HCV infection was 0.22 per 100 patient-years (PY) in the cohort. The incidence rate increased from 0.13 per 100PY before 2002 to 0.19 per 100PY in 2002–2007 and 0.47 per 100PY in 2008–2013. All the seroconverters were Chinese, with median age of anti-HCV seroconversion at 38 years (range: 28–53 years). None of them were injecting drug users. As compared with the non-seroconverters, seroconverters were of higher education level (85.7% vs 50.7% tertiary education or above, OR 5.28, p=0.021) and had prior history of sexually transmitted infection (92.9% vs 60.9%, OR 8.34, p=0.041). More seroconverters were found to have history of syphilis infection (57.1% vs 37.2%, p=0.134) but the difference was not statistically significant. Baseline CD4 count and HIV viral load, proportion on antiretroviral therapy and duration of antiretroviral therapy were not different between two groups.

**Conclusions:**

The incidence of HCV has been increasing among HIV-infected MSM non-injecting drug users in Hong Kong. More education and intervention on safer sex is required to be targeted on those with higher education level.

**Table 1:** Comparison between HCV seroconverters and HCV non-seroconverters

		HCV seroconverters (n = 14)	HCV non-seroconverters (n = 1297)	p
Median age (years)		38	40	0.556
Ethnicity	Chinese	14 (100%)	1126 (86.8%)	0.996
	Non-Chinese	0	171 (13.2%)	
Education level	No school or below secondary school	2 (14.3%)	639 (49.3%)	0.021
	Tertiary school or above	12 (85.7%)	658 (50.7%)	
Baseline median CD4 count (μl^3^)		261	330	0.331
Baseline median HIV viral load (cp/mL^3^)		935,000	55,000	0.509
On antiretroviral therapy		9 (64%)	833 (64.2%)	0.996
Median duration of antiretroviral therapy (days)		317	479	0.962
Prior history of sexually transmitted infection		13 (92.9%)	790 (60.9%)	0.041
Prior history of syphilis		8 (57.1%)	482 (37.2%)	0.134

